# The efficacy of ocular surface assessment approaches in evaluating dry eye treatment with artificial tears

**DOI:** 10.1038/s41598-022-26327-3

**Published:** 2022-12-17

**Authors:** Dorota H. Szczesna-Iskander, Maria Muzyka-Wozniak, Clara Llorens Quintana

**Affiliations:** 1 Department of Optics and Photonics, Wroclaw University of Science and Technology, Wybrzeze Wyspianskiego 27, 50-370 Wroclaw, Poland; 2 Ophthalmology Clinical Centre SPEKTRUM, Wroclaw, Poland; 3 Research and Development Centre, CREO, Wroclaw, Poland; 4 Casey Eye Institute and Department of Ophthalmology, Oregon Health and Science University, Portland, OR USA

**Keywords:** Biomedical engineering, Diagnosis, Eye manifestations

## Abstract

This study evaluates the effectiveness of objective techniques in assessing dry eye disease (DED) treatment compared with the subjective assessment commonly used in clinical practice. Thirty subjects were recruited for two visits separated by 28(± 3) days of treatment with artificial tears. A buttery of common subjective assessment methods were accompanied by a set of objective techniques including measurement of noninvasive tear film break-up time (NIBUT), lipid layer thickness (LLT), and quantitative evaluation of tear film surface quality and dynamics (TFD). Additionally, meibography was performed. Two commercially available videokeratoscopes and a prototype of a lateral shearing interferometer were used for the measurements. Both subjective and objective techniques showed a positive effect of artificial tears in DED treatment. Statistically significant improvements were observed in subjective symptoms (from *P* < 0.001 for Ocular Surface Disease Index, OSDI to *p* = 0.019 for tearing), conjunctival redness (*P* = 0.022), ocular staining (*P* = 0.012), fluorescein tear film break-up time (*P* = 0.015), NIBUT (*P* = 0.037), LLT (*P* < 0.001), and TFD (*P* = 0.048). In general, weak or statistically insignificant correlations were observed between subjective and objective assessment methods. The apparent lack of correlation between these methods might indicate the complementary character of objective techniques that likely assess other characteristics of ocular surface health than those assessed subjectively.

## Introduction

The Tear Film and Ocular Surface Society Dry Eye Workshop II (DEWS II) defines dry eye disease (DED) as a multifactorial disorder that cannot be characterised by a single sign or symptom^1^. Two main subtypes of DED including evaporative dry eye, affected by lipid anomaly, and aqueous-deficient dry eye can be recognized, but they usually coexist. Differentiating and determining the dominant DED type is clinically challenging. Blepharitis, Meibomian gland dysfunction (MGD), and ocular surface inflammation add to this sometimes foggy picture^2,3^.

The diagnosis of DED usually comprises a combination of subjective complaints assessed with different types of questionnaires and signs that are subjectively observed on slit-lamp biomicroscopy. Ocular surface staining assessment, fluorescein tear film break-up time (FBUT), and Schirmer test, which are often used in clinical practice, are invasive since they affect the natural tear film stability and secretion, and their results are difficult to assess objectively^4^. According to the DEWS II Report^5^, a noninvasive approach is preferable for diagnosing DED. In particular, noninvasive tear film break-up time (NIBUT) has been proposed as an objective and repeatable tool for tear film stability assessment.

The number of videokeratoscopes measuring NIBUT has been steadily increasing. Each device has a specific, often proprietary software to estimate NIBUT from the distortion of Placido rings reflection from the pre-corneal tear film. The results may vary between devices depending on the technique and software used, but recently, a unified approach has been proposed^6^. The numerical analysis of each recorded frame that presents the temporal stage of tear film surface quality (TFSQ) provides the opportunity to evaluate the dynamics of TFSQ between blinks^7^. These dynamics differ between healthy and pathological tear film^8^, therefore, more parameters describing tear film stability than the single NIBUT estimate are available.

MGD is the most common cause of DED^9^. Apart from the subjective assessment of lid margin and meibum, the diagnosis of DED subtype can be additionally supported by assessing lipid layer interference fringes (i.e. lipid layer thickness (LLT)) and Meibomian gland (MG) morphology. Many investigations have emphasized the need for more exhaustive research to improve the correlation of ocular imaging, including MGs, with clinical subjective assessments in DED^10–12^. There is a consensus that the combination of both morphological and functional evaluation of MG would be essential to provide further insights into the DED pathophysiology. Practitioners subjectively evaluate MGs and LLT using grading scales. Although MGD quantification techniques’ advance, none of the methods has been validated^13^.

The study aimed to test the efficacy of noninvasive, objective techniques in diagnosing DED and to monitor the results of DED treatment with commonly used ocular lubricant, and further to compare the results with those derived from frequently used slit-lamp examinations and the evaluation of subjective symptoms. In addition, the association of previously proposed tear film surface measures^6,14,15^ and commonly used tear film parameters was investigated. The hypothesis of the study is that the objective measures of tear film dynamics correlate with traditional measures of tear film stability and quality and both should show improvement after applied treatment.

## Results

The study enrolled 30 Caucasian subjects (27 women), with a mean age of 52 years (SD ± 15 years; range 20–81 years). None of the subjects reported noncompliance with eye drops use. Seventeen subjects reported office work using a computer. Six subjects were regular smokers and six subjects underwent cataract surgery at least 6 months before the study. None of the subjects had a known autoimmune disease or allergy, nor were current contact lens wearer. Except for two subjects with an OSDI score < 22 who were diagnosed with mild dry eye, the subjects were diagnosed with severe dry eye. According to the above-mentioned criteria, twenty-six (87%) subjects were diagnosed with aqueous deficient dry eye (ADDE). All subjects had MGD-related dry eye with either abnormal morphological lid features, meibum quality, or meibomian gland dropout above or equal to grade 1 in the lower and/or upper eyelid. The results of all the measurement methods except for MG morphology, were compared before and after treatment.

Mean temperature and relative humidity in the examination room were 25.6 ± 2.1 °C and 43.4 ± 11.2%, respectively, at Visit 1 and 23.4 ± 1.2 °C and 34.3 ± 8.3% at Visit 2. A statistically significant reduction in room temperature (*P* < 0.001) and relative humidity (*P* = 0.001) was noted at Visit 2.

No significant difference between the first and second measurement of tear film parameters was observed (*P* > 0.05). Table 1 presents the median and range values of the clinical parameters evaluated at both visits, together with the results of Wilcoxon test (*P*-value). Statistically significant improvement was noted in some symptoms, including subjective feeling assessed with the OSDI questionnaire, which decreased significantly from moderate to mild. All subjects had some corneal staining that was reduced by at least 1 point in 19 of the subjects, and 23 subjects (77%) had some conjunctival staining at Visit 1 that was also significantly reduced at the Visit 2. All subjects with bulbar conjunctiva redness grade 2 or 1 at Visit 1 presented no conjunctival redness at Visit 2. Although TMH assessed in slit-lamp did not show significant change, the TMH assessed with K5M increased significantly after treatment. 23 subjects (73%) who had an LLT score of 2 or less (LLT of 30 to 50 nm or less) had an increase in LLT at Visit 2. All methods of tear film break-up time evaluation showed longer times after treatment. However, only FBUT and NIBUT_K5M showed statistically significant improvement.

**Table 1:** Median and [range values] of the clinically assessed signs and symptoms before (Visit 1) and after (Visit 2) treatment.

Test	Visit 1		Visit 2		*P*-value
	Median	Range	Median	Range	
**Symptoms**					
Itchy eye [scores]	1.0	[0.0–3.0]	0.0	[0.0–2.0]	0.004*
Tearing [scores]	1.0	[0.0–2.0]	0.0	[0.0–2.0]	0.019*
Discomfort [scores]	2.0	[0.0–3.0]	0.0	[0.0–3.0]	< 0.001*
Discharge [scores]	0.0	[0.0–2.0]	0.0	[0.0–3.0]	0.590
Photophobia [scores]	2.0	[0.0–2.0)	1.0	[0.0–2.0)	0.300
OSDI [scores]	41.3	[13.4–72.0]	23.8	[2.1–71.3]	< 0.001*
**Subjective/invasive assessment**					
Bulbar conjunctiva redness [scores]	1.0	[0.0–2.0]	0.0	[0.0–2.0]	0.022*
Bulbar conjunctiva oedema [scores]	1.0	[0.0–2.0]	1.0	[0.0–2.0]	0.160
MG secretion [scores]	1.0	[0.0–2.0]	1.0	[0.0–2.0]	0.362
Lid margin hyperaemia [scores]	0.5	[0.0–2.0]	0.0	[0.0–0.1]	0.119
Corneal staining [scores]	2.0	[1.0–3.0]	1.0	[0.0–2.0]	< 0.001*
Conjunctival staining [scores]	1.0	[0.0–3.0]	0.5	[0.0–3.0]	0.012*
Schirmer test [mm]	6.5	[0–15]	6	[2–25]	0.394
TMH [slit-lamp] [mm]	0.20	[0.10–0.50]	0.20	[0.05–0.70]	0.743
FBUT [s]	4.5	[1.0–9.7]	6.3	[1.0–21.0]	0.015*
LLT [scores]	2.0	[1.0–3.0]	3.0	[1.0–4.0]	< 0.001*
**Objective/noninvasive assessment**					
TMH [K5M software ruler] [mm]	0.25	[0.14–0.55]	0.32	[0.17–0.67]	0.011*
NIKBUT_first [s]	7.46	[3.06–20.84]	8.13	[2.40–23.26]	0.770
NIBUT_K5M [s]	8.53	[1.63–16.54]	10.48	[1.00–22.02]	0.037*
NIBUT_E300 [s]	7.06	[1.00–23.92]	11.35	[1.00–23.92]	0.098
TFSQ_K5M [a.u.]	15.80	[0–34.55]	12.99	[0–33.13]	0.327
TFSQ_E300 [a.u.]	43.60	[25.88–109.81]	41.01	[28.92–107.60]	0.846
TFSQ_LSI [a.u.]	117	[103–161]	114	[108 136]	0.227
TFD_K5M [slope]	3.11	[**− **74.49–77.10]	4.00	[**− **45.71–62.46]	0.480
TFD_E300 [slope]	35.00	[**− **41.60–75.01]	37.66	[**− **33.39–73.28]	0.405
TFD_LSI [slope]	0.80	[**− **0.45–8.37]	0.23	[**− **1.37–3.89]	0.048*

Statistically significant differences are marked with asterisk.

Bold *P*-values indicate p < 0.05 between visits assessed with Wilcoxon test.

[a.u.]—arbitrary unit.

*OSDI* ocular surface disease index, *MG* meibomian glands, *FBUT* fluorescein break-up time, *TMH* tear meniscus height, *LLT* lipid layer thickness, *NIKBUT* noninvasive keratograph break-up time, *NIBUT* noninvasive break-up time, *TFSQ* tear film surface quality, *LSI* lateral shearing interferometer, *K5M* keratograph 5M (Oculus), *E300* keratograph (medmont).

Fitting bilinear or linear function to the TFSQ time course revealed that the median of TFSQ average level was lower after treatment meaning better TFSQ, in all three noninvasive techniques, but this change was not statistically significant. TFD measured by LSI under natural blinking conditions (TFD_LSI) showed significant improvement in tear film stability.

Table 2 presents the median of results obtained for automated analysis of MG morphology in the upper eyelids and the meiboscore for the lower eyelid. 47% of the subjects had a meiboscore of 0 or 0.5, 13% of 1.0 or 1.5, 33% of 2.0 or 2.5 and, only one subject of 3.0. There was one subject with zero meiboscore in both eyelids, however she had abnormal meibum quality assessed as grade 2.0 and higher Meibomian glands irregularity compared to the group median. Median gland length shows shortening of 44% of the total length.

**Table 2:** Characteristic of Meibomian glands morphology in the upper eyelid.

Feature	Median (range)
Meiboscore (lower eyelid)	1.0 (0–3)
Meiboscore grade	1.5 (0–3)
Gland atrophy area	32.5% (0–68)%
Average number of glands	18 (4–25)
Irregularity of glands	30% (3–58)%
Gland length	2.1 mm (1.4–3.8) mm
Gland width	0.4 mm (0.3–0.6 mm)
Standard deviation of gland length (variability)	1.0 mm (0.7–2.2) mm
Standard deviation of gland width (variability)	0.2 mm (0–0.3) mm

**Figure 1:**
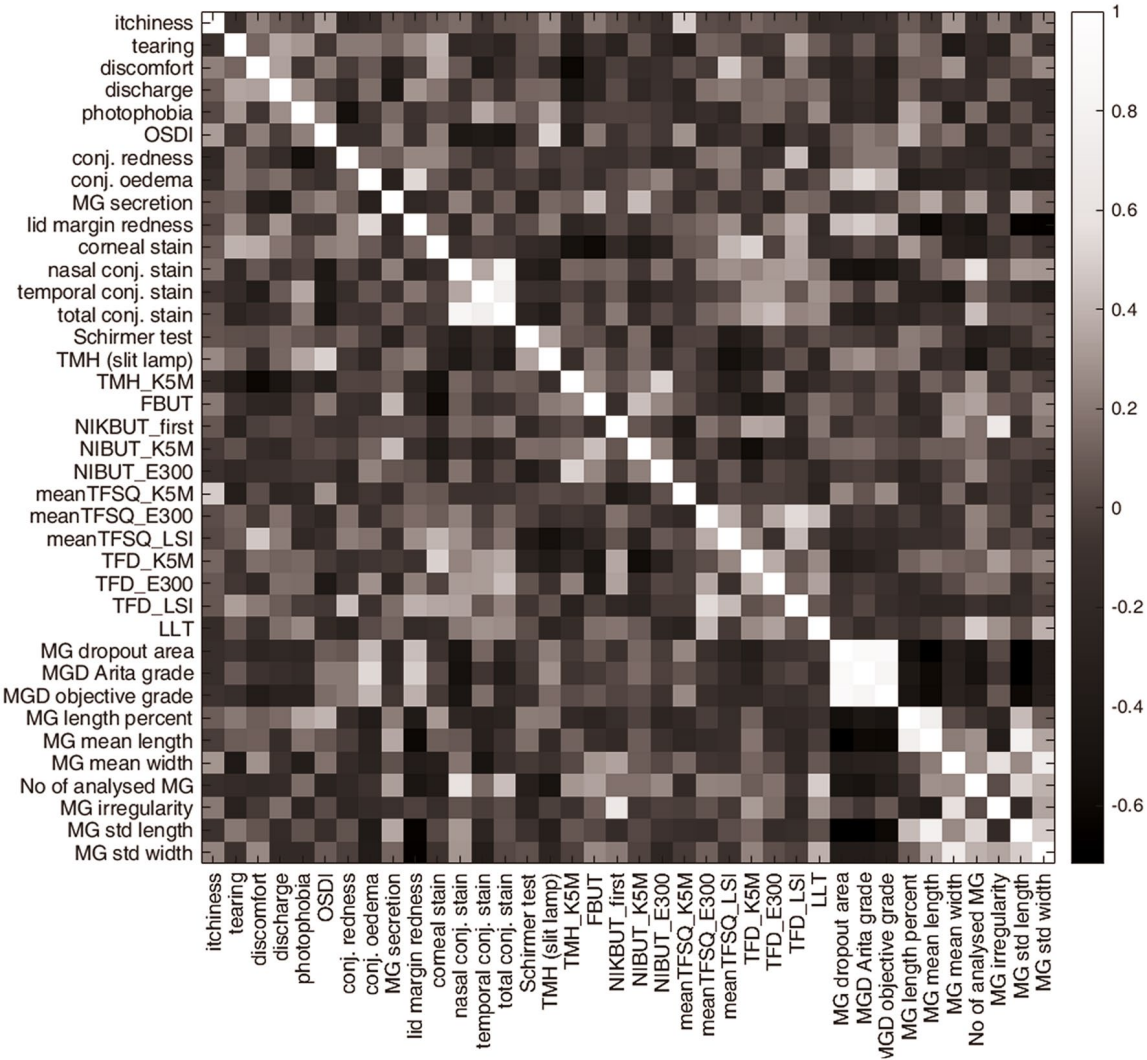
The matrix of results of all calculated correlations. The *r*-value is graded from black, the lowest obtained value (*r* = − 0.65), to white, *r* = 1.

Figure 1 shows the results for all calculated correlations and Table 3 shows the statistically significant correlations (for clarity, only those with *P* < 0.01 are shown and *r* values close to the threshold of 0.489 set for the sample size of 30). Partial correlation calculated for the differences between visits (i.e., a parameter at Visit 2 minus that parameter at Visit 1) with eye drop type as control revealed moderate correlation (*P* < 0.01) only between decreased conjunctival staining and longer NIBUT_K5M (*r* = 0.491, *P* = 0.007).

**Table 3:** Statistically significant correlations (*P* < 0.01) between all (pair-wise) considered parameters for Visit 1.

Symptoms	Subjective/objective assessment	*r*	*P*-value
Itchy eye	TFSQ_K5M	**0.489**	0.006
Discomfort	TMH_K5M	**− 0.561**	0.001
Discomfort	TFSQ_LSI	0.480	0.007
**Subjective assessment**	**Objective assessment**		
Conjunctival redness	TFD_LSI	0.468	0.009
MG secretion	TMH_K5M	–0.409	0.002
Corneal staining	TFD_K5M	0.482	0.007
Corneal staining	FBUT	–0.488	0.006
**Subjective/objective assessment**	**MG objective assessment**		
Bulbar conjunctiva oedema	Automatically assigned Arita grade (upper eyelid)	**0.535**	0.007
Lid margin hyperaemia	MG mean length	**− 0.605**	0.002
Lid margin hyperaemia	Std length	**− 0.644**	0.001
Lid margin hyperaemia	Std width	**− 0.651**	0.001
Conjunctival staining nasal	Automatically assigned Arita grade (upper eyelid)	**− 0.536**	0.007
Conjunctival staining nasal	Subjectively assessed Arita grade (lower eyelid)	**− 0.532**	0.007
Conjunctival staining nasal	No of analysed glands	**− 0.574**	0.003
NIKBUT_first	MG irregularity	**0.669**	< 0.001
LLT	No of analysed glands	**0.542**	0.007

Bold font indicates significant non-zero correlations at 5% level of significance and 80% power for the given sample sizes of 30 (i.e., those ≥ 0.489).

Bold p-values indicate a partial correlation with *P* < 0.01.

*MG* meibomian glands, *FBUT* fluorescein break-up time, *TMH* tear meniscus height, *LLT* lipid layer thickness, *NIKBUT* noninvasive keratograph break-up time, *NIBUT* noninvasive break-up time, *TFSQ* tear film surface quality, *LSI* lateral shearing interferometer, *K5M* keratograph 5 M (oculus), *E300* keratograph (medmont).

## Discussion

Reduction of ocular irritation symptoms is commonly observed after DED treatment with topical lubricating products regardless of the regularity of dosing^16^, even if other assessed parameters of tear film homeostasis do not reveal significant improvement^17–20^. Here, simple lubrication helped to improve DED symptoms within 28 ± 3 days.

Diagnosing DED is challenging because of its multifactorial aetiology and variability of signs and symptoms. Each tear film layer plays an important role in maintaining a stable tear film. The Rasch analysis, used for the evaluation of single estimate validation of dry eye severity, showed ocular surface staining as the most informative indicator^21^. In our study, corneal and conjunctival staining, bulbar conjunctiva redness, and FBUT also significantly improved. These results agree with those of Wang et al.^22^ and Lee et al.^23^, who assessed the effectiveness of artificial tears treatment after one month of use. It is concluded that the better condition of the ocular surface is linked to the longer tear film stability observed after treatment.

Since the emphasis is on the development of noninvasive diagnostic methods, a continuing trend is observed in instruments assessing the ocular surface to incorporate modalities for noninvasive and automated evaluation of tear film stability^13^. Objective methods are needed to document the effectiveness of DED therapy^21^. It is known, that FBUT may be influenced by the amount of fluorescein used^24^, while noninvasive, automated measurements obtained with a videokeratoscope are expected to provide unbiased results. All methods used in this study showed longer tear film break-up time after treatment (mean differences of 2.73 ± 5.14, 0.53 ± 5.39, 2.32 ± 5.05 and 3.07 ± 9.56 s, for FBUT, NIKBUT_first, NIBUT_K5M and NIBUT_E300, respectively). Although the unified approach of image analysis was applied to calculate NIBUT in order to overcome the bias introduced by different image analysis techniques, only the customized analysis applied for K5M revealed significantly longer NIBUT after treatment in comparison to other noninvasive assessments, specifically, in comparison to the built-in software of the K5M. This confirms the difficulty of maintaining similar measuring conditions, and that the results are influenced by the properties of each instrument. In addition, tear film stability depends on blink type and potential subject’s fatigue. Although care has been taken to minimize such factors by asking subjects to blink naturally and allowing two-minute breaks between measurements as they might have some effect on the variability of the results^25^. Additionally, TFSQ has inherent variability owing to its cyclical behavior, where sudden changes in the lipid patterns may occur between two blinks after mixing with the reservoir lipids^26^. This may affect the quality of reflection of the Placido rings from the tear film surface, which cannot be controlled. In other studies, where aqueous-based artificial tears were used for DED treatment over a similar time period (i.e., 30 days), tear film stability assessed with fluorescein showed a significant improvement^17,22^. Similar results were obtained when NIBUTs were assessed using Placido rings-based instruments^18–20^.

This study showed that artificial tears improved tear film surface dynamics assessed with LSI. It is also important to note that post-blink deterioration is less dynamic, and the slope sign was negative in many cases at Visit 2, meaning that it takes longer than the first second after blinking to smooth out the tear film surface.

Many studies showed a discordance between symptoms and signs of DED^27–30^. In this study, a few statistically significant correlations were found between the signs and symptoms. A stable tear film and evenly spread lipid layer provide a smooth and regular surface on the cornea. The quality of vision drops dramatically if this surface becomes irregular^31^, being an additional factor to discomfort. This may explain the correlation between the average TFSQ_LSI and subjective overall comfort, which may be also related to the need for more frequent blinking and blurred vision.

No correlation between ocular surface condition and tear film break-up, with or without fluorescein, was observed in this study. In addition, the estimates of the invasive and noninvasive tear film break-up times did not correspond with subjective feeling. Nevertheless, significant correlation between corneal staining and bulbar conjunctival redness and tear volume (TMH_K5M) with tear film dynamics was noted, showing a more stable tear film after treatment.

Attention was paid to the time of Visit 2 after the application of the drops, as it is important to perform tear film measurements at similar time of the day as well as after a similar time from the last eye drops instillation. Recommendations by Paugh et al.^32^ and Schmidl et al.^33^, were followed and measurement were performed two to four hours from the last artificial drop instillation.

The study limitation was unstable environmental conditions. The significant difference in temperature and relative humidity between visits (it was cooler and less humid at Visit 2) could be the possible reason for the lack of significant improvement in most of the tear film stability test results, including TFD and mean TFSQ. Although the differences in average temperature and relative humidity were small (2.3 °C and 9.1%, respectively), the measurement environment may have affected the tear film break-up time^34^.

In a population-based study, the MGD symptoms did not correlate with FBUT or fluorescein staining^30^. However, lissamine green conjunctival staining may be present earlier in DED than corneal fluorescein staining^35^. Significant correlations between the MG meiboscore grade of the lower and upper eyelids and nasal conjunctival lissamine green staining were found, as well as with number of glands. As MGD progresses, MGs become irregular, tortuous and atrophic. Loss of MG results in a thinner lipid layer, and we found a significant correlation between number of visible glands and LLT, indicating that at thicker lipid layer was observed in eyes with more visible glands. However, LLT did not correlate with any other MG parameters as expected. One possible reason for this is the subjectivity of LLT assessment. One could expect that when the LLT is thinner, the tear film stability is compromised. In this study, no significant correlations were observed between any of the tear film stability test results, except for NIKBUT_first, which was correlated with MG irregularity. This suggests a more important role of the lipid layer structure, such as polar lipids^8,26^, than its thickness in tear film stability.

The negative correlation between lid margin hyperaemia and mean MG length indicated that the shorter the glands are, the worse the condition of the lid margin is. This result is accompanied by a significant negative correlation between lid margin hyperaemia and the standard deviation of the length and width of the glands, which means that subjects with worse conditions of the lid margin also have greater variability in the shape of the glands—not all glands have been shortened yet.

Arita’s meiboscore to assess MG atrophy was positively correlated only with the bulbar conjunctival oedema score. This agrees with other studies that did not find any correlation between MG loss and Schirmer test or tear film break-up time^36,37^. The objectively assessed MG parameters did not correlate with the symptoms assessed using the OSDI questionnaire which has also been observed previously^36,38,39^.

An alternative tear volume measurement to the Schirmer test is the tear meniscus height, which has the advantage of being noninvasive. The digitally and objectively assessed TMH_K5M increased after treatment, although no significant change was detected in TMH assessed with a slit-lamp. Therefore, the objectively measured TMH_5KM also showed a negative correlation with discomfort and eye itchiness, manifesting higher diagnostic potential than the slit-lamp TMH or Schirmer test.

In summary, corneal staining and discomfort were the main clinical parameters that significantly improved after treatment and correlated with some objective measures. Among all the objective indices, significant improvement after treatment was observed for LLT, TMH_K5M, NIBUT_K5M, and TFD_LSI. Of these, only TMH_5KM was correlated with corneal staining and discomfort. Corneal staining also correlated with TFD_K5M, although the change in TFD_5KM after treatment was not statistically significant.

The assessment of lipid layer, NIBUT based on videokeratoscopy, and tear film dynamics based on interferometry may be considered as tear film biomarkers that have the potential to monitor DED treatment. However, despite advanced digital analysis of tear film dynamics, only the least sophisticated parameter among the considered, TMH_5KM, seemed to judiciously measure the ocular lubricant effect on tear film status. Unfortunately, variability and dynamic changes in tear film behavior seem to compromise the effectiveness of more advanced assessment methods. The dynamic nature of tear film and the impact of many factors on its behavior such as blink type, lipid layer cycle are the reason why averaging several consecutive measurements across the appointment is a necessity to compare the results from different techniques and visits. Averaging two measurements of tear film dynamics in this study may not be sufficient to obtain statistically significant correlations with traditional measures of tear film stability and quality, and to notice a significant improvement in all considered objective measures after applied treatment.

Summarizing, the hypothesis of the study was that noninvasive and objective methods of tear film imaging and numerical analysis correlate with subjective and qualitative assessments of ocular surface health, and with this correlation, the effect of treatment could be measured using methods that in previous studies have shown sensitivity to subtle changes in the surface of the tear film. The results of the experiment showed that this hypothesis was not confirmed.

## Patients and methods

### Study design

This was a randomized prospective comparative study. The study was conducted in compliance with the principles of the Declaration of Helsinki and approved by the local ethics committee of the Lower Silesia Medical Council (4/BOBD/2018). Written informed consent was obtained from all participants before enrolment. The subjects were patients from the Ophthalmology Clinical Centre SPEKTRUM, Wroclaw, Poland, where the study was conducted and their ophthalmic history reviewed.

After the 7-day wash-out period, the subjects were treated with commercially available artificial tear formulation, containing sodium hyaluronate 0.24%, natrium chloride, disodium phosphate decahydrate, sodium dihydrophosphate dehydrate, and sterile water.

During the treatment period of 28 days (± 3 days) subjects were instructed to self-administer one drop of the artificial tears on the surface of both eyes four times a day. No additional treatments, such as eyelid warming or massage, were allowed throughout the study period. Subjects were asked to maintain high compliance and to use the drops of not less than two and not more than four hours before the assessment.

A sample size of twenty-five individuals ensures 90% power at a 5% level of significance to detect a 1 s difference between the measurements based on FBUT and NIKBUT variability. The sample size requirement for correlation *r* ≥ 0.500 being significantly different from the null correlation of zero at 80% power and 5% level of significance is 29^40^.

### Subjects

Eligible participants were adults (aged > 18 years) who had self-reported history of DED and fulfilled the following three inclusion criteria: Ocular Surface Disease Index (OSDI) score > 13, FBUT < 10 s and significant staining of cornea and/or conjunctiva > 1 assessed with the Ocular Staining Score (OSS)^41^. Exclusion criteria were pregnancy, diabetes, any known allergies, history of ocular or periocular infection within prior two months, history of laser vision correction or any other ocular surgical procedures within prior six months, or any continuous use of eye drops other than artificial tears.

### Measurement protocol

**Figure 2:**
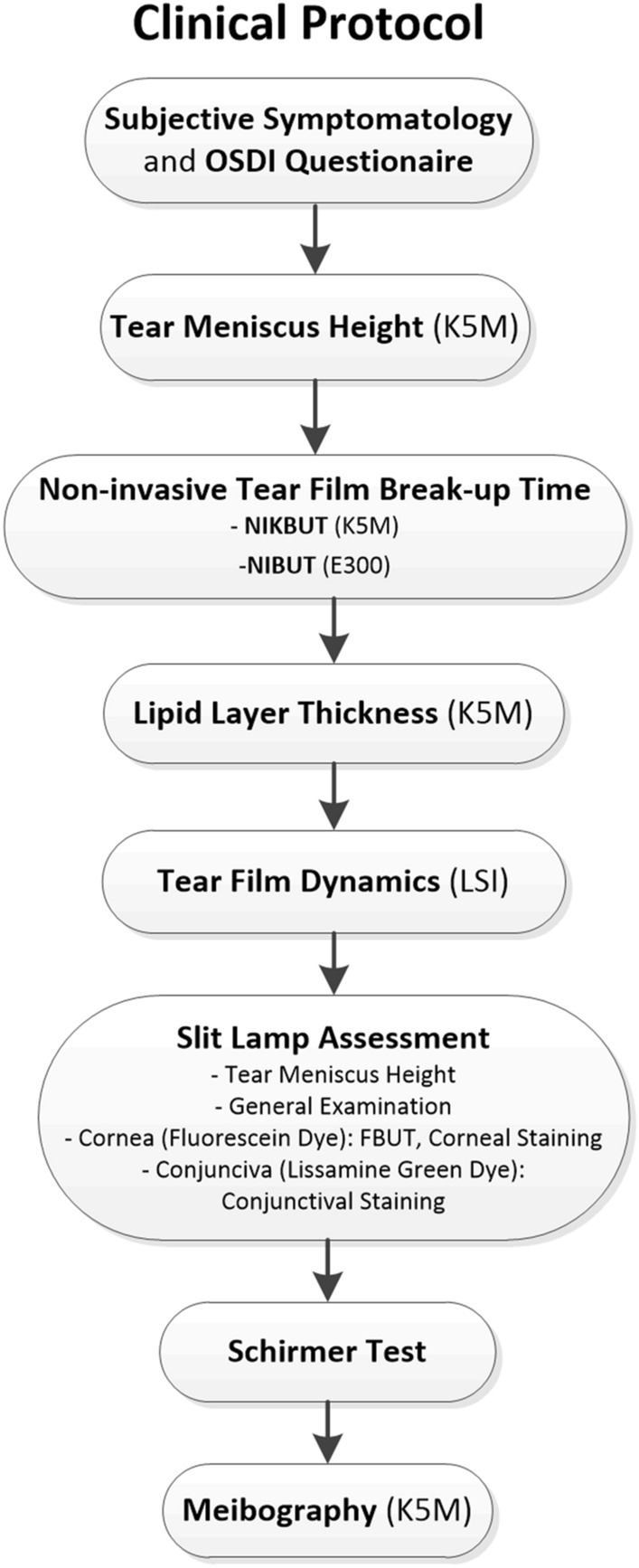
Flow diagram of the study protocol.

All assessments were performed at Visit 1 (baseline) and Visit 2 (control after treatment) in the same standardized order established according to DEWS II recommendations and starting with the least invasive methods. Figure 2 presents the measurements order and a more detailed description is provided below. All clinical assessments were performed by the same experienced ophthalmologist (M.M.-W.) in the same examination room. All measurements were performed before and after DED treatment, except for MG morphology, which was only measured at Visit 1 because no visible change within one month was assumed. In order to limit the “chair time” and to conform to the guidelines for statistical analysis^42^, only right eye was evaluated although the left eye was also assessed in slit-lamp to confirm the DED diagnosis.

#### Symptomatology assessment

Subjective assessments included OSDI^43^ and subjective symptoms records. OSDI was completed by subjects without assistance. Additionally, subjective symptoms such as itching, tearing, discomfort (i.e., foreign body sensation, eye burning and pinching), discharge and photophobia, were graded depending on severity on a four-step scale (grade 0–3, see Table 4).

**Table 4:** The description of symptoms provided for the subjects.

	Grade 0	Grade 1	Grade 2	Grade 3
Itching	No need to rub the eye	Occasional need to scratch	Frequent need to scratch	Constant need to scratch
Tearing	Normal amount of tears	Increased amount of tears without (emotional) tearing	Periodic tearing	Constant tearing
Discomfort	None	Mild	Moderate	Severe
Discharge	None	Small amount	Eyelids sticky in the morning	Strong morning clump of the eyelids
Photophobia	None	Slight difficulties in light requiring squinting	No significant difficulties requiring the wearing of sunglasses	Inability to withstand natural light even with sunglasses

#### Tear film volume

Tear film volume was assessed based on tear meniscus height (TMH) photographed with Keratograph 5 M (K5M) (Oculus Optikgeräte, Wetzlar, Germany). Digital calipers were used to measure TMH in the central lid margin. Additionally, slit-lamp biomicroscope was used to compare TMH with the calibrated slit-lamp beam height.

#### Noninvasive tear film surface quality and stability

**Figure 3:**
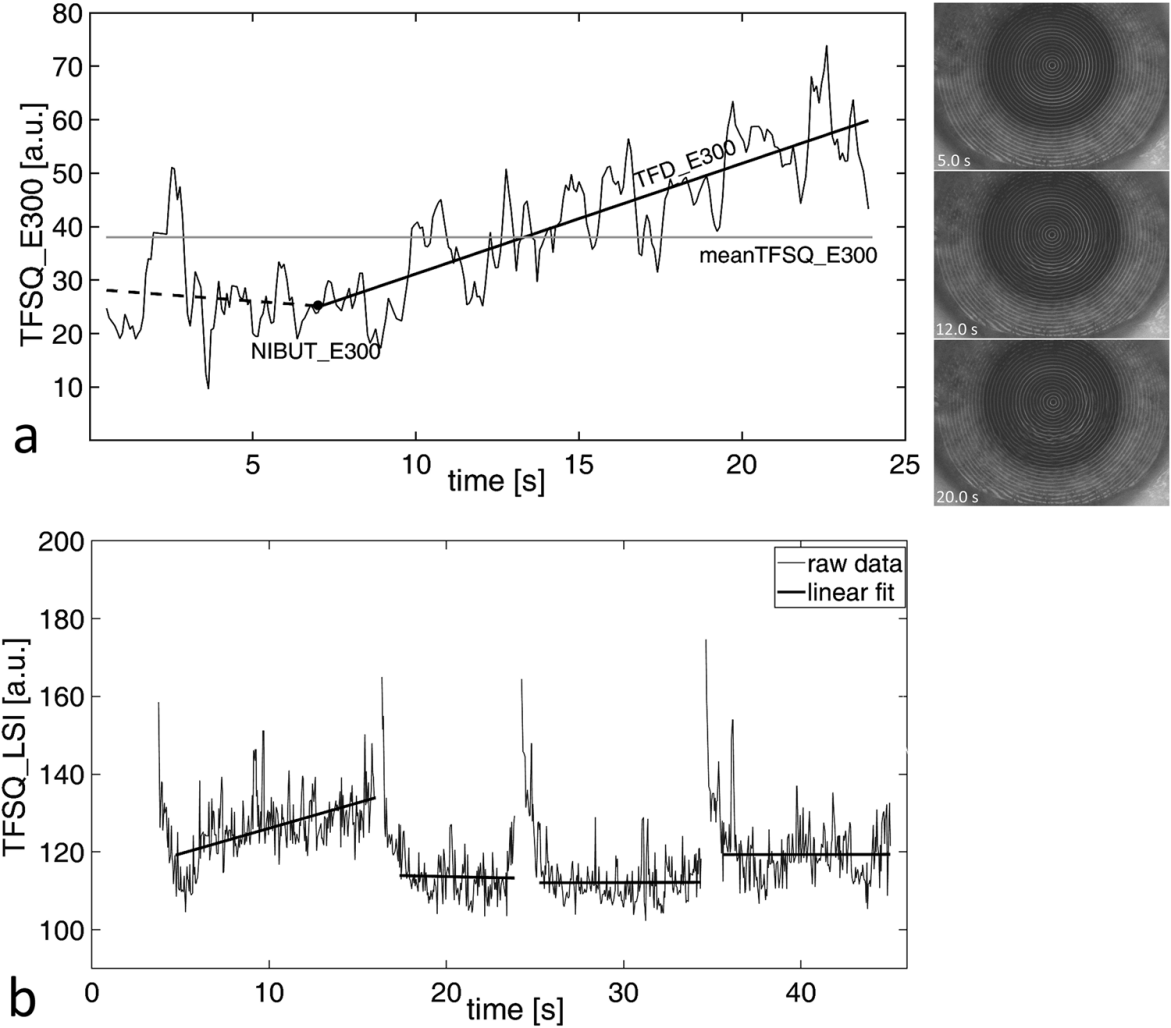
Time series of tear film surface quality (TFSQ). (**a**): Example of TFSQ assessment by E300 where the dashed line represents the stable phase of the tear film and the solid line represents the destabilization phase of the tear film (tear film dynamics, TFD_E300). The black dot represents an estimate of TFSQ at the break-up time as the start of tear film instabilities (NIBUT_E300). The average TFSQ is calculated along the entire inter-blink interval (TFSQ_E300). The sample frames on the right illustrate the instantaneous state of Placido rings after the blink. (**b**) Example of TFSQ assessment by LSI. Solid lines are the linear fit to the data of each interblink (omitting the first second).

Noninvasive tear film imaging was performed using two videokeratoscopes, K5M and E300 (Medmont Pty., LTD, Melbourne, Australia) and the lateral shearing interferometer (LSI), a prototype designed for assessing tear film surface quality^14^. The K5M software was used to calculate the keratograph tear film break-up time (NIKBUT_first) as the time between a blink and the first appearance of deformation of Placido rings reflection. E300 and LSI were used to assess TFSQ under suppressed and natural blinking conditions, respectively. Additionally, K5M and E300 raw videos were further exported and analyzed using a custom-written algorithm that estimates the regularity of a Placido rings image based on estimating its fractal dimension, as described earlier^44^. Briefly, the algorithm analyzed the morphological changes in the reflected Placido rings (using either videokeratoscope). This allowed unifying the tear film analysis across the two instruments. According to this analysis lower TFSQ values correspond to a less distorted ring pattern (smoother tear film) and higher TFSQ values correspond to a more distorted reflected pattern. Due to the dynamic nature of the tear film, when estimating NIBUT, it is important to find the time point at which tear film stability begins to deteriorate. Figure 3a shows an example of the measurement outcome, where the break-up time is the point at which the TFSQ trend starts to increase (NIBUT_E300). This point was determined by the constrained fit of two linear functions^14^. The slope of the second line indicates tear film dynamics (TFD), denoted TFD_E300 in Fig. 3a, when the deterioration of Placido rings increases gradually in time. This increasing distortion of Placido rings reflection is depicted in the illustrative frames (Fig. 3a right).

Two measurements were performed with both videokeratoscopes separated by two minutes. The average of each parameter was taken for further analysis.

With LSI, in contrast to the videokeratoscopy, subjects were asked to blink as naturally as possible. For each subject, an image sequence of 40 s was recorded with a sampling frequency of 25 Hz. The interference of wavefronts reflected from the tear film surface indicates its smoothness. Numerical analysis of interferograms describe each image in the recorded sequence with a number^7^ that defines the temporal estimate of TFSQ. Two parameters were estimated from the time sequences obtained from the linear fitting of each interblink interval data, 1 s after blink (see Fig. 3b): the average value of TFSQ—meanTFSQ_LSI, and the slope (trend of change in TFD—TFD_LSI). These two parameters were averaged for the entire sequence within several interblink intervals recorded within each measurement sequence.

TFSQ increase when the tear film surface starts to deteriorate, and tear film break-ups become more evident^7,44^. Lower values of TFSQ indicate smoother tear film surface and lower values of TFD indicate more stable tear film (values close to zero) or tear film levelling process (negative values).

#### Lipid layer thickness

LLT was assessed with K5M using white light interferometry. The color interference fringes were recorded for 30 s while the subject was blinking naturally. Then, the same masked clinician subjectively graded all acquired videos using the 4-category grading scale proposed by Remeseiro et al.^45^, (levels 1 to 4, from thinnest to thickest lipid layer).

#### Slit-lamp assessment

Dry eye diagnostic examinations were performed using slit-lamp biomicroscopy and included the assessment of bulbar conjunctiva redness, oedema, lower lid margin redness, FBUT, corneal fluorescein staining, and conjunctival lissamine green staining. MG secretion was assessed using slit-lamp after the Schirmer test. It was performed by applying digital pressure to eight central glands of the lower eyelid and was graded between 0 and 3: 0, clear meibum readily expressed; 1, cloudy meibum with mild pressure; 2, cloudy meibum expressed with more than moderate pressure; 3, meibum could not be expressed.

To measure FBUT a drop of saline solution was placed on fluorescein strip (Madhu Instruments Pvt. Ltd. New Delhi, India). The strip was applied to the temporal canthal lid margin area. The slit-lamp magnification was set at × 10; cobalt blue light and a Wratten 12 yellow filter were used to enhance observation of tear film. The time between blink and the first appearance of a dark spot was recorded using a stopwatch. FBUT was measured three times and averaged. Corneal staining was assessed two minutes after FBUT assessment.

A drop of saline solution was placed on a lissamine green 1% ophthalmic strip (BIOTECH Vision Care Pvt. Ltd., Gujarat, India) allowing soaking into the strip for at least 5 s. A drop of dye was then applied to the temporal canthal lid margin area. Nasal and temporal conjunctival staining was assessed three minutes after instillation. The Ocular Staining Score was used to grade both corneal and conjunctival staining severity^46^.

#### Schirmer 1 test

Schirmer 1 test was performed after applying topical anaesthesia with 0.5% proxymetacaine hydrochloride eye-drops (Alcaine, Alcon). Schirmer test ophthalmic strips (OPTITECH Europe BVBA, Gingelom, Belgium) were inserted into the lower conjunctival sac at the junction of the lateral and middle thirds and the wetted length of the strip in millimeters was recorded after five minutes.

#### Meibomian gland morphology

The K5M module for MG assessment with infrared light illumination was used to obtain images of inverted upper and lower eyelids. Evaluation of gland dropout and morphology of the upper eyelid was performed with an automated objective analysis using a customized algorithm^15^. The analysis provides gland morphology descriptors including the percentage of drop-out area with respect to the total exposed tarsal conjunctiva, the mean length and width of the glands, mean percentage length of the glands with respect to the amount of exposed tarsal conjunctiva, the number of glands, and the mean gland irregularity described as a percentage of how different the gland shape is from a regular gland (the higher the percentage, the greater the irregularity), understanding a regular gland as an elongated elliptical-like shaped gland along the tarsal conjunctiva. Standard deviations of the gland mean length and width were also assessed. Furthermore, the percentage of area with gland atrophy was classified according to the scale proposed by Arita et al. (i.e., meiboscore)^47^, where grade 0 means no gland atrophy (area of drop-out 0%); grade 1, 2 and 3 (area of gland atrophy < 33% 33–67% and > 67%, respectively). As this algorithm has been validated for the upper eyelid, MGs morphology of the lower eyelid was assessed subjectively and graded using Arita’s grading scale^47^, because the subjects with evaporative DED present more alterations in the lower lid than healthy subjects^48^.

#### Dry eye subtype

The predominant DED subtype, i.e., aqueous deficiency (ADDE) or evaporative (EDE) was defined based on slit-lamp biomicroscopy and meibography assessment. The ADDE subtype was indicated if a scant inferior tear meniscus was < 0.2 mm or Schirmer test was < 10 mm. The EDE subtype was indicated in the presence of capped, plugged or atrophied MGs, cloudy, frothy or absent MG secretion upon digital expression of the lower MGs and lid margin redness. The diagnosis of MGD-related disease was additionally considered in accordance with the MGD Report^49^ as follows: the grade of morphologic lid features or the expressibility/quality of meibum was above 1 or the meiboscore was at least grade 1 in the lower or upper eyelid.

### Statistical analysis

The Jacque-Berra test was used to test the data for the hypothesis of normality. The statistical significance of differences between baseline results (Visit 1) and the results after treatment (Visit 2) were assessed with Wilcoxon test. Spearman correlation coefficient was used to evaluate the relationships between all pairs of considered parameters at Visit 1. Partial correlation was used to assess the relationships between visit differences for all parameters. For all the tests, the significance level was set at 5%.

## Data Availability

Full datasets generated and analyzed during the current study are not publicly available as public data sharing was not included in the ethics committee approval but are available from the corresponding author on reasonable request.
